# ‘Becoming a Nurse’: A Lifelong Journey Beyond the Transition to Professional Practice‐An Analytical Discursive Paper

**DOI:** 10.1111/jan.70630

**Published:** 2026-05-02

**Authors:** Seung A. (Sarah) Park, Marion Eckert, Rebecca Sharp, Judy Duchscher, Micah D. J. Peters

**Affiliations:** ^1^ Rosemary Bryant AO Research Centre, College of Health Adelaide University Adelaide South Australia Australia; ^2^ College of Nursing and Health Sciences Flinders University Adelaide South Australia Australia; ^3^ School of Nursing and Midwifery, College of Health Adelaide University Adelaide South Australia Australia; ^4^ School of Nursing & Population Health Building Thompson Rivers University Kamloops British Columbia Canada; ^5^ Australian Nursing and Midwifery Federation (Federal Office) Melbourne Victoria Australia; ^6^ Health Evidence Synthesis, Recommendations, and Impact (HESRI), School of Public Health, College of Health Adelaide University Adelaide South Australia Australia; ^7^ The Danish Centre of Systematic Reviews: a JBI Centre of Excellence, Department of Clinical Medicine Aalborg University Aalborg Denmark; ^8^ Office of the Pro‐Vice Chancellor—First Nations Education and Research University of Southern Ipswich Queensland Australia; ^9^ School of Nursing and Midwifery Edith Cowan University Joondalup Western Australia Australia

**Keywords:** career stages, career transition, lifespan development, nurse, nursing, nursing education, professional identity, transition theory, workforce development, workforce sustainability

## Abstract

**Aim:**

To reconceptualise becoming a nurse as a lifelong developmental journey that extends beyond the traditional focus on new graduate transition, and to inform policies and practices supporting recruitment, retention and career sustainability.

**Background:**

The global nursing shortage persists, exacerbated by high attrition rates among new graduates and an aging workforce. While existing literature predominantly examines the transition from student to professional practice, we propose that ‘becoming a nurse’ begins earlier and extends beyond clinical roles into retirement.

**Design:**

An analytical discursive paper.

**Data Sources:**

Key theoretical frameworks (Schlossberg's Transition Model, Kennedy's Integrated Transition Model, Benner's Novice‐to‐Expert framework and Duchscher's Stages of Transition Model) were integrated with empirical literature (1974–2025) on nursing career trajectories, clinical expertise development and professional identity across the lifespan, with emphasis on contemporary evidence from 2015 to 2025.

**Results:**

Becoming a nurse is reconceptualised as a continuous, lifelong transition encompassing four interrelated phases: (1) early career interest, where nursing aspirations emerge during childhood and adolescence; (2) non‐traditional entry, involving second‐career entrants who undergo profound identity reconstruction as novices; (3) middle‐career transition, characterised by sustained development from competence through proficiency toward expertise and clinical wisdom; and (4) late‐career transition, where professional identity and contribution continue beyond retirement. Each phase presents distinct developmental demands requiring tailored educational, organisational and workforce responses. Together, these phases form a Lifespan Transition Framework that advances the field by proposing transition as a recursive developmental mechanism, where adaptive capacities built at each phase become foundational resources for subsequent phases, rather than separate, time‐limited events.

**Conclusion:**

Understanding becoming a nurse as a lifelong transition provides a unifying conceptual foundation for more coherent, stage‐sensitive workforce strategies. This perspective shifts policy and practice beyond short‐term graduate retention toward lifespan‐oriented workforce systems that strengthen recruitment, sustain expertise and preserve professional wisdom across the whole nursing career lifespan.

## No Patient or Public Contribution

This analytical discursive paper synthesises theoretical frameworks and empirical literature to reconceptualise nursing transition across the professional lifespan. As a conceptual analysis drawing on published scholarship and workforce data, it did not involve direct participation of patients, service users, caregivers, or members of the public in its design, conduct, or interpretation.

## 1 Introduction

The global nursing crisis persists, with the World Health Organization (2025) forecasting a 4.1 million worker shortfall by the end of the decade. Nursing workforce shortages are widespread, and although this human resource challenge has been documented for nearly a century, we have yet to comprehensively address their root causes (Peters 2023). The COVID‐19 pandemic further strained workforce retention, with many jurisdictions reporting exacerbated challenges to attracting and retaining nurses (ICN 2021; Sharplin et al. 2025). A contributing factor to this workforce crisis is the challenging transition period experienced by new graduate nurses, which is well documented as exceptionally disruptive, characterised by heightened stress, job dissatisfaction and increased attrition (Casey et al. 2021). To understand the complexities of this phenomenon, scholars have long examined the transition experiences of new graduate nurses (Kramer 1974; Duchscher 2009). While the transition of new graduates into professional practice has received considerable attention, this focus may obscure a more comprehensive reality: becoming a nurse is a lifelong journey that begins well before university and continues beyond retirement. Overlooking this broader view could risk missed opportunities to develop effective workforce strategies for recruitment, retention and professional identity across the career lifespan. Considering global nursing shortages, increasingly diverse entry pathways into the profession, and the evolving complexity of healthcare systems, a more nuanced understanding of how nurses enter, navigate and sustain their careers is urgently needed. Broadening our understanding of when the journey to becoming a nurse begins could also enable the development of effective strategies to tap into a previously overlooked and upstream reservoir of future nurses.

This paper discusses the concept of transition to practice in nursing as a continuous, cyclical process rather than a finite, linear event. Drawing on theoretical perspectives including Schlossberg's Transition Theory, Kennedy's Integrated Transition (KIT) Model, Duchscher's Transition Shock Model and Benner's Novice to Expert Framework, we explore how transition can be understood to manifest during four key phases: early career interest (e.g., school leavers), non‐traditional entry (e.g., second‐career entrants who enter as nursing novices), middle‐career transition (e.g., the evolution from competence to expertise and clinical wisdom), and late‐career transition (e.g., nursing career reinvention). Importantly, regardless of when or how individuals enter nursing, whether directly from school or after established careers in other fields, all practitioners begin as nursing novices who embark on a lifelong, continuous transition journey, progressively developing discipline‐specific clinical judgement, pattern recognition and practice wisdom through sustained experience over decades (Benner 1984). The broadened perspective of becoming a nurse can inform educational design, workforce strategies and professional development initiatives throughout one's nursing career. This paper, therefore, advances a lifespan‐based reconceptualisation of becoming a nurse as a continuous developmental process, rather than a time‐limited early‐career adjustment. We propose the Lifespan Transition Framework, conceptualising transition as a recursive developmental mechanism across four interdependent phases, such that adaptive capacities developed in one phase become resources for subsequent phases.

## 2 Background

Despite ongoing and diverse mitigation efforts, nursing workforce shortages have intensified into a persistent global structural crisis (Peters 2023). According to the State of the World's Nursing 2025 report, the global nursing pool has expanded to 29.8 million; however, significant investment is still needed to bridge a projected 4.1 million worker deficit by the end of the decade. This effort is critical as 19% of the workforce prepares for retirement amid rising global health demands (World Health Organisation [WHO] 2025). Expanding on this global picture, considerable disparities in the scale and distribution of the nursing workforce exist across Europe. Recent WHO reporting shows nurse‐and‐midwifery personnel density ranging from 27.0 per 10,000 population in Türkiye to 183.7 per 10,000 in Switzerland and 202.7 per 10,000 in Monaco, highlighting persistent regional imbalances in workforce capacity (WHO Regional Office for Europe 2023). Comparable pressures are also evident in other high‐income regions. In the United States, an estimated 800,000 nurses are expected to leave the profession by 2027, further straining an already overburdened healthcare system (Martin et al. 2023). A comparable trend is evident in Australia, where registered nurses and midwives account for 55% of the 642,479 registered health practitioners. The Australian Department of Health anticipates a shortfall of 85,000 nurses by 2025 and 123,000 by 2030, driven in part by persistently high turnover rates (Health Workforce Australia 2014; Department of Health 2021).

This workforce crisis is not confined to high‐income nations. Countries such as the Philippines and India, which have traditionally been major contributors to the global nursing supply, are now facing acute domestic shortages due to high emigration rates and limited local employment opportunities (Press Trust of India 2024; The Star 2025). In India, the nurse‐to‐population ratio stands at just 1.96 per 1000, falling short of the World Health Organisation's recommended minimum of 3 per 1000 (Press Trust of India 2024). Similarly, while the Philippines produced 37,098 newly registered nurses in 2024, a substantial proportion were expected to migrate abroad or transition to non‐nursing careers due to low domestic wages and restricted career advancement (The Star 2025). These global trends highlight a systemic, transnational issue that demands sustainable and long‐term strategies to improve nurse retention and workforce resilience (Buchan and Catton 2023).

The COVID‐19 pandemic has further exacerbated this crisis, placing an unprecedented strain on nurses' well‐being and undermining retention efforts (ICN 2021). These retention vulnerabilities must be understood not as isolated pandemic effects but as the amplification of longstanding structural fragilities within nursing transition systems. New graduate nurses, typically defined as those within the first 12 to 24 months of practice, entering the workforce during this volatile period, faced high levels of stress and dissatisfaction, resulting in alarming rates of attrition (Casey et al. 2021). During this stressful period, new graduates frequently reported a dissonance between their initial idealism and the realities of clinical practice, leading to negative effects on retention due to diminished job satisfaction (Phuekphan et al. 2021). Empirical evidence supports these findings: a longitudinal study in Shanghai involving 343 new graduate nurses found that 17.2% planned to resign shortly after starting work. This percentage increased to 59.6% after four months and peaked at 74.4% by 8 months, reflecting the considerable strain experienced during the early phase of the continuous transition to professional practice (Zhang et al. 2017).

To understand the complexities of this phenomenon, scholars have long examined the transition experiences of new graduate nurses, notably through Kramer's concept of ‘reality shock’, which was later refined by Duchscher into the ‘transition shock’ model (Duchscher 2009; Kramer 1974). Duchscher's Stages of Transition (Duchscher 2008) capture the emotional, cognitive and professional dissonance experienced by new nurses at three stages: ‘Doing (1–3 months), Being (4–8 months) and Knowing (9–12 months)’. Transition shock is experienced by the newly graduated nurse during Duchscher's initial stage of transition and embodies the rather volatile, dynamic and highly emotionally charged period of time immediately after graduation and once their workplace orientation has concluded (Duchscher 2009). Support mechanisms, such as mentorship, preceptorship and transition‐to‐practice programs, have been implemented to aid this adjustment and reduce attrition (Ankers et al. 2018; Gularte‐Rinaldo et al. 2023). Nevertheless, concerns remain regarding the adequacy of these supports. For instance, a mixed‐methods study of newly graduated nurses found that, despite feeling generally supported within the transition program, participants witnessed concerning negative behaviours, including bullying, and experienced workplace support challenges that hindered their development of competence and confidence, potentially leading to job dissatisfaction (Charette et al. 2023). These findings were echoed in Kenny et al.'s (2021) systematic review, which found that structured preceptorship, mentorship and organisational support enhance the transition experience of graduate nurses.

While the existing literature provides valuable insights into the post‐graduation transition, it may not fully capture the complexity of the professional journey. The concept of ‘becoming a nurse’ should be viewed as a broader, more dynamic process that is neither confined to nor fully defined by the immediate post‐graduation period. Instead, this journey begins much earlier, often when individuals first consider nursing as a career, and continues well beyond formal employment.

### 2.1 Researcher Reflexivity and Positionality

This discussion paper was developed through a collaborative, interdisciplinary approach that integrates perspectives from nursing education, social science and workforce policy. The authorship team includes nurse academics (RS, SP) with extensive experience teaching across undergraduate nursing curricula from entry to graduation, who contributed practical insight into how professional identity, preparedness and capability evolve across educational and early practice contexts. A social scientist and anthropologist (MP) provided a critical sociocultural lens for interpreting nursing as a lifelong, developmental journey rather than a discrete early‐career transition. The conceptual framing was further informed by an internationally recognised professional role transition theorist (JD) and a workforce innovation leader (ME) with expertise in health services research and workforce sustainability. This diversity of perspectives enabled the conceptual extension of existing transition models across the professional lifespan.

### 2.2 Data Sources

This discursive paper draws on multiple sources of evidence to construct a comprehensive understanding of ‘becoming a nurse’ as a lifelong process.

Theoretical frameworks: Schlossberg's Transition Theory (Schlossberg 2005), Kennedy's Integrated Transition (KIT) Model (Musamali 2018), Duchscher's Stages of Transition Theory (Duchscher 2008) and Benner's Novice to Expert framework (Benner 1984) provide complementary perspectives on transition processes, identity formation and professional development across career stages.

Empirical nursing literature: MEDLINE, Embase, CINAHL and Google Scholar searches (1974–2025) identified studies examining nursing career trajectories and transition experiences. Studies were selected based on conceptual relevance to understanding nursing professional development beyond the traditional graduate transition period.

Career development research: Literature (2009–2024) examining childhood career aspirations, adolescent career decision‐making and factors influencing late‐career workforce participation extends understanding of the broader life‐course perspectives.

Workforce and policy documents: Global and national workforce data were drawn from the World Health Organization (2025), WHO Regional Office for Europe (2023), the International Council of Nurses (2021), the Australian Department of Health (2021), the Australian Institute of Health and Welfare (2024) and Health Workforce Australia (2014). Additional labour market insights were sourced from national media reporting, including the Press Trust of India (2024) and The Star (2025).

### 2.3 Overview of Issues

The prevailing nursing transition scholarship continues to be dominated by a time‐limited conceptualisation of transition, most commonly defined as the first 12 months following registration (Duchscher 2008). While this body of work, often grounded in the concept of ‘transition shock’ (Duchscher 2009), has generated critical insights into early‐career vulnerability and graduate attrition, it may also implicitly assume that the transition is understood as an early‐career adjustment rather than as a continuous, cumulative and lifelong phenomenon, which we argue is more organically and practically reflective and relatable in terms of how people grow, evolve and change over the course of a lifetime.

We pose that the conceptual limitation of focusing on a rigidly ‘time‐bound’ notion of becoming a nurse is increasingly misaligned with contemporary workforce realities, where it is often said that a person will change career between three and seven times in a lifetime (The University of Queensland 2023). Career aspirations often form well before university when individuals first consider nursing as a career. Second‐career entrants undergo complex identity reconstruction as they shift from expert status in one field to novice status in nursing, and their professional capabilities continue to evolve (Benner 1984). However, these transitions are rarely theorised as part of a coherent lifespan process. Further, the most extended and professionally transformative phase of nursing, the middle career transition, remains theoretically under‐integrated within transition scholarship. Over decades of professional practice, nurses move from competence toward proficiency and, for some, toward expert and wise practice. This extended developmental process underpins patient safety, system efficiency, leadership capacity and workforce stability, yet it is rarely conceptualised as a form of transition despite being alluded to in requirements to engage in continued professional development activities and recency of practice requirements as components of formal registration standards in many jurisdictions (Pool et al. 2016; Price and Reichert 2017; Vázquez‐Calatayud et al. 2021). Similarly, many nurses maintain strong professional identities and continue contributing through mentorship, leadership and advisory roles long after leaving clinical employment, despite workforce frameworks typically positioning retirement as an endpoint rather than as a further phase of professional transition (MacLeod et al. 2021).

Collectively, these gaps highlight a deeper conceptual limitation: transitions in nursing have largely been theorised as a series of discrete episodes rather than as a developmental process unfolding across the entire professional lifespan. Although existing frameworks, such as Schlossberg's Transition Model, Kennedy's Integrated Transition Model, Benner's Novice‐to‐Expert framework and Duchscher's Stages of Transition/Transition shock, provide valuable insights into how individuals navigate change, build expertise and reconstruct professional identity, their use has predominantly been restricted to specific points in the career trajectory, particularly the new graduate period. By extending existing transition frameworks from early‐career interests through to late‐career transitions, this paper adopts an integrated lifespan transition lens to reposition professional identity, expertise and workforce sustainability as cumulative and interdependent processes. Our novel contribution is the explicit integration of these frameworks into a single explanatory Lifespan Transition Framework, distinguishing transition as a recursive developmental mechanism (a cumulative developmental process) rather than a simple temporal extension of early‐career adjustment.

Lifespan perspectives on transition are also evident in the broader career and retirement literatures. For example, Akkermans et al. (2024) synthesise longitudinal evidence on career transitions across the lifespan and propose a self‐regulation lens to integrate diverse transition types. Likewise, Zhan et al. (2019) apply lifespan‐development perspectives to the work‐to‐retirement transition, using person–environment fit to explain how planning, decision‐making and adjustment unfold in later career. However, these works do not provide a nursing‐specific, phase‐structured framework of becoming a nurse that integrates nursing transition theories across the full professional arc (from pre‐professional interest formation through post‐retirement contribution). Accordingly, the Lifespan Transition Framework proposed here is not a re‐labelling of existing lifespan perspectives; it is a profession‐specific conceptual synthesis that specifies four interdependent phases and the recursive developmental mechanism linking them.

## 3 Findings

### 3.1 Transition Models: Frameworks for Understanding the Journey of Becoming a Nurse

Understanding the journey of becoming a nurse requires more than focusing solely on the transition from student to graduate. Several theoretical models offer valuable insights into this journey, highlighting that transitions in nursing are continuous, multifaceted and socially embedded processes. Four influential frameworks inform this discursive synthesis: Schlossberg's Transition Model, Kennedy's Integrated Transition (KIT) Model, Benner's Novice‐to‐Expert framework and Duchscher's Stages of Transition Theory and Transition shock. Each framework illuminates different aspects of the nursing transition experience across time and context.

*Schlossberg's Transition Model* provides a comprehensive framework for understanding how individuals navigate transitions, emphasising their continuous and adaptive nature (Schlossberg 2005). Transitions are conceptualised as ongoing processes of appraisal and assimilation, shaped by both anticipated and unanticipated events. The model identifies four key factors influencing an individual's ability to manage transitions: *Situation* (the context and conditions surrounding the transition); *Self* (personal attributes and resources individuals draw upon to cope with change); *Support* (external resources, including mentorship and organisational structures); and *Strategies* (approaches and plans employed to address transitional challenges). These interacting elements provide insight into how individuals respond to change across multiple life stages.

*Kennedy's Integrated Transition (KIT) Model*, grounded in constructionist theory, frames transitions as dynamic and socially constructed processes shaped by human interactions and environmental contexts (Musamali 2018). The model outlines a cyclical process with two interrelated phases: *Adjustment*, described as a period of disruption and reorganisation as individuals navigate unfamiliar roles and responsibilities; and *Adaptation*, a stage of integration and internalisation in which new practices and identities are consolidated within a transformed context. The KIT Model emphasises the role of social interaction and environmental factors in shaping transitions, positioning transition as a lifelong phenomenon applicable across career stages (Harrison et al. 2020).

*Benner's Novice‐to‐Expert Framework* describes the progressive development of clinical competence, illustrating how experience and learning contribute to skill acquisition and professional growth in nursing practice (Benner 1984). The model outlines five levels of skill proficiency: *Novice* (nursing students with minimal or no clinical experience); *Advanced Beginner* (new graduates who have completed their education and gained some experience through clinical placements); *Competent* (nurses who prioritise tasks and make decisions based on prior experience but may struggle with stress and adaptability in complex situations); *Proficient* (nurses who develop an intuitive and flexible approach, anticipate problems and respond to subtle cues with improved clinical judgement); and *Expert* (highly experienced nurses who operate with deep tacit knowledge and clinical intuition). While Benner's Novice‐to‐Expert framework is not a transition theory, it provides valuable insight into how clinical competence progressively evolves from early practice to later stages through accumulated clinical experience.

*Duchscher's Stages of Transition Theory* builds upon Kramer's (1974) original concept of reality shock, with specific focus on the experiences of new graduate nurses entering the workforce (Duchscher 2008). It captures the non‐linear and dynamic nature of professional role transition within three overlapping stages: *Doing* (1–3 months), characterised by feelings of inadequacy and overwhelming workplace expectations, during which transition shock is typically most intense; *Being* (4–8 months), marked by gradual adaptation and growing awareness of workplace realities; and *Knowing* (9–12 months), a consolidation phase where theoretical knowledge increasingly aligns with practical experience. *Transition shock*, coined by Duchscher (2009), is a conceptual construct that explains the intensity and disruption commonly experienced during early professional transitions. Transition shock is a multidimensional response encompassing emotional distress, physical exhaustion, cognitive dissonance and sociocultural dislocation that arises from the discrepancy between educational preparation and the realities of clinical practice. While most pronounced during the early doing stage, transition shock is not confined to a single point in time; rather, it reflects the cumulative impact of increased responsibility, accountability and professional socialisation within complex healthcare environments. The degree and duration of transition shock are influenced by organisational context, support structures, role clarity and workload demands (Duchscher 2009). Although the concept has predominantly been applied to the first year of practice, its emphasis on disruption, adjustment and identity reformation suggests broader relevance to other transition points across a nurse's professional trajectory.

Collectively, these four frameworks provide a multidimensional lens for examining nursing transition across time. While they have historically been applied most often to early career stages, each offers conceptual elements that can be extended across broader phases of the professional lifespan.

### 3.2 Early Career Interest: Formation of Nursing Aspirations Before University

The term transition originates from the Latin transition, meaning ‘a going across or over’, suggesting that transition is a process of personal transformation over time (Harper n.d.). In the context of nursing, this perspective reveals that the journey to becoming a nurse often begins long before university enrolment, yet this early phase remains underexplored within the nursing literature.

The international *Drawing the Future survey*, involving over 7000 children aged 7–11 across 20 countries, found that career aspirations are primarily shaped by immediate role models and media exposure (Chambers et al. 2018). In Australia, 45% of children know someone, such as a parent or teacher, working in their desired profession, while 47% rely on television, film, social media, or radio to inform their ambitions when direct exposure is limited (Chambers et al. 2018). Globally, children in developing countries tend to aspire to practical roles, such as teaching or medicine, whereas those in developed nations are more influenced by celebrity culture. For instance, 38% of Australian children aspire to become sportspeople compared with only 8% expressing interest in healthcare careers (Chambers et al. 2018). This disparity is notable given healthcare's status as Australia's fastest‐growing sector, with a projected 25% increase in employment between 2017 and 2022 (Australian Government Department of Employment 2017).

Professional identity in nursing is significantly shaped by initial encounters with nurses and healthcare environments, which influence individuals' perceptions of the profession, their self‐identification with nursing attributes, and ultimately their career decisions (Price 2009). During adolescence, career aspirations, including those for nursing, consolidate as secondary school students explore healthcare pathways and envision future professional roles (Godbey and Gordon 2019). In Victoria, Australia, government‐led initiatives introduce formal career exploration during the first years of lower secondary school (Years 7 and 8), enabling students aged 12–14 to identify their values, strengths and vocational interests. By Year 9, programs such as *My Career Insights*, facilitated by the Australian Centre for Career Development, offer tailored counselling and assessment tools such as the Morrisby Profile (Victorian Department of Education 2024).

Career decision‐making during adolescence is influenced by personal reflection and broader sociocultural factors, including media portrayals, school‐based messaging and personal experiences with healthcare (Godbey and Gordon 2019). Nursing, however, is often misrepresented through outdated and narrow media portrayals that fail to capture its intellectual demands, autonomy and professional complexity (González et al. 2023). Such stereotypes can portray nursing as less prestigious than other health professions, discouraging high‐achieving students from pursuing it in favour of medicine, physiotherapy, or veterinary science (Raymond et al. 2018). These perceptions are reflected in workforce trends, with allied health and medicine growing by 67% and 41%, respectively, between 2013 and 2022, compared with only 26% growth in nursing and midwifery (Australian Institute of Health and Welfare 2024). While these trends reflect systemic workforce patterns, they also indicate the limited visibility of diverse nursing roles in public discourse and career education (Raymond et al. 2018).

### 3.3 Non‐Traditional Entry: Second‐Career Entrants as Nursing Novices

The process of becoming a nurse is not always linear or confined to early adulthood. An increasing number of individuals with tertiary qualifications in other disciplines are entering nursing as a second career through graduate‐entry programs, motivated by a desire for meaningful work, job stability and career progression (Macdiarmid, McClunie‐Trust, et al. 2021). This pathway is particularly relevant in the context of ongoing global nursing shortages, with mid‐career entrants offering a potential means to diversify and strengthen the workforce (World Health Organization 2025). Terminology describing career‐change pathways varies internationally. In Australia and New Zealand, graduate entry broadly refers to individuals entering nursing with a prior degree (McKenna and Vanderheide 2012). In North America, more specific program titles are used: Accelerated Bachelor of Science in Nursing programs award a Bachelor of Science in Nursing over 12–18 months, while Entry‐Level Master of Science in Nursing programs award a master's qualification over 24–36 months (American Association of Colleges of Nursing 2023). For consistency, this paper uses the term ‘graduate‐entry programs’ as an umbrella term for these pathways.

The development of graduate‐entry nursing programs in the United States during the 1970s represented a strategic response to workforce shortages, offering accelerated pathways for individuals with non‐nursing degrees (McKenna and Vanderheide 2012). Similar initiatives have emerged in Australia, including programs such as the Master of Nursing Science, which enable individuals from diverse academic and professional backgrounds to achieve registration within two years (Macdiarmid, McClunie‐Trust, et al. 2021). These accelerated programs contribute to workforce sustainability and have positioned nursing as an attractive second career for individuals motivated by altruistic values, job security and personal experiences with healthcare as patients, caregivers, or volunteers (Macdiarmid, Turner, et al. 2021). They may also appeal to individuals seeking career trajectories beyond direct clinical practice, including policy, regulation, research, education and health leadership. Second‐career entrants bring valuable attributes from their prior careers, including professional maturity, communication skills, emotional intelligence and heightened responsibility (Nursing World Org 2023).

Traditional undergraduate nursing students begin their professional journey at the novice stage, progressively developing foundational knowledge, technical skills and professional identity through structured coursework and supervised clinical placements and transition to advanced beginner upon graduation (Larson et al. 2013; Ozdemir 2019). Second‐career entrants likewise commence nursing practice as novices, despite bringing valuable skills and perspectives from previous careers and may experience developmental challenges similar to those of traditional undergraduate nursing students. Mohamed (2019) highlights that second‐career entrants frequently report higher levels of stress and burnout as they form a new professional identity while managing established life responsibilities. The phenomenon of failure to rescue, reported as high as 37% among novice nurses, underscores that safe and effective clinical judgement is discipline‐specific and fundamentally grounded in situated nursing experience (Saintsing et al. 2011; Benner 1984). In addition to these shared developmental demands, individuals from culturally and linguistically diverse backgrounds may face further challenges in adapting to the profession's norms, communication styles and expectations (Dos Santos 2020; Colley et al. 2023). These challenges are not only academic but also social and cultural, with potential implications for student retention, academic progression and professional integration.

Despite their prior life and professional experience, second‐career entrants remain vulnerable to the same early‐practice risks, learning curves and emotional demands that shape the transition experiences of traditional new graduates. To address these complexities, educational institutions could implement flexible program structures, such as part‐time and blended learning, and strengthen recognition of prior learning to enhance confidence and academic progression (Kean et al. 2022).

### 3.4 Middle Career Transition: From Competence to Expertise and Wisdom

Nurses who successfully navigate the initial transition period enter a consolidation phase in which growing experience fosters organised, efficient and anticipatory practice. With sustained engagement, some progress toward expert marked by tacit knowledge, refined intuition, and advanced situational awareness (Benner 1984). At this level, nurses can anticipate problems before they fully manifest, recognise when standard approaches may not apply to particular patients, and adapt interventions with nuance and precision (Gartshore et al. 2024).

Beyond expertise, contemporary nursing scholarship increasingly conceptualises clinical wisdom or wisdom‐in‐action as a qualitatively different state of practice that emerges through extensive reflective engagement (Matney et al. 2020). Clinical wisdom reflects the integration of extensive experiential knowledge with moral sensitivity, contextual awareness and what is often described as nursing's sixth sense (Matney et al. 2020). The Theory of Wisdom‐in‐Action for Clinical Nursing describes how wise nurses practise using their head, their heart and their gut, applying general wisdom processes in stressful or uncertain situations while developing personal wisdom through reflection, meaning discovery and learning (Matney et al. 2020).

This evolved practice wisdom is evident in advanced clinical roles. Without suggesting that nurse practitioners (NP) universally represent the pinnacle of nursing wisdom, as indeed NPs will also evolve in the same manner as other nurses and indeed other clinicians, as an advanced nursing role requiring additional education and experience beyond that of a registered nurse, NPs do serve as a prime example. Systematic reviews of nurse practitioner (NP) care demonstrate that NPs provide care equal to or better than physicians across multiple patient and health system outcomes (McMenamin et al. 2023). Evidence shows that NP‐led care is associated with reduced or similar costs, equivalent or better quality and similar or lower rates of emergency department use and hospitalisation compared with models without NP involvement (McMenamin et al. 2023). These outcomes illustrate how the cumulative development of nursing expertise enhances system efficiency and patient outcomes.

The middle career transition also involves profound growth in nurses' collaborative capacity and organisational influence. Experienced nurses develop sophisticated understandings of how healthcare systems function, enabling them to work effectively across professional boundaries, advocate strategically for patients and contribute to system‐level improvement (Al Hajri 2024). This is also evident in the way nurses have taken on leadership positions in government, research, policy, legal practice and business. Even those who remain in direct bedside roles frequently serve as informal leaders, role models and change agents, identifying safety gaps, mentoring colleagues and shaping unit culture (Pool et al. 2016). Their accumulated practice wisdom becomes a critical organisational asset, supporting quality improvement, patient safety and system resilience (Goens and Giannotti 2024).

Situated within the broader transition continuum, the middle years represent the most extended and most professionally transformative phase of becoming a nurse (Price and Reichert 2017). The progression from novice to advanced beginner, competent, proficient, expert and ultimately toward clinical wisdom cannot be accelerated through education alone but requires sustained, deliberate and reflective practice over time (Pool et al. 2016; Patel and Metersky 2022).

### 3.5 Late‐Career Transition: Professional Identity Beyond Clinical Employment

For many nurses, formal employment ends without equating to disengagement from the profession. Instead, many remain actively involved through mentorship, research, advisory roles and community‐based initiatives, reaffirming that nursing is not merely an occupation, but a core aspect of personal and professional identity developed over many years (MacLeod et al. 2021). As MacLeod et al. (2021) note, sentiments such as ‘First and foremost, I am a nurse’ (p. 5) reflect the enduring professional identity of these nurses.

Nurses aged 55 and older are commonly categorised as being in the late‐career transition phase; however, many continue to work, by choice or necessity, into their 60s, 70s and 80s. As retirement ages and life expectancies increase, this pattern is expected to become more common globally. Despite this, with one in six nurses worldwide expected to retire within the next decade, the potential loss of institutional knowledge, clinical judgement and mentorship capacity presents a significant challenge to the global nursing workforce (World Health Organization 2025; Cimino‐Fiallos and Wolff 2022). Many late‐career nurses view retirement as a transition to redefined professional roles aligned with evolving capacities and preferences rather than an endpoint (MacLeod et al. 2021).

Late‐career nurses often encounter structural and systemic barriers to continued engagement. The physically demanding nature of clinical work, including manual handling, prolonged standing, irregular shift work and disrupted sleep patterns, can become increasingly burdensome with age, particularly when accompanied by chronic health conditions or fatigue (Denton et al. 2021a). Workplace conditions, including limited role diversity, a lack of perceived respect and insufficient opportunities for career progression, may also contribute to early retirement decisions (Australian College of Nursing 2023). At the policy level, leadership development for senior nurses remains inconsistent. While 82% of countries report having senior nursing officers responsible for workforce development, only 25% of low‐income countries provide structured leadership training, exacerbating global disparities and underutilisation of this workforce segment (World Health Organization 2025).

Strategic interventions to support continued engagement include ergonomic modifications to reduce physical workloads, flexible scheduling and formal mentorship structures to facilitate intergenerational knowledge transfer (Gan 2020; Australian College of Nursing 2023). Recognition through awards, titles, or leadership roles can further affirm the ongoing value of experienced nurses and enhance engagement and satisfaction (Pagone and Briggs 2021). As Denton et al. (2021b) emphasise, leveraging late‐career nurses' practice‐based wisdom can significantly enhance workforce capability and organisational resilience.

## 4 Discussion

### 4.1 Conceptual Contribution

sThis paper advances nursing transition scholarship by proposing a Lifespan Transition Framework articulated through three novel theoretical propositions that extend beyond existing models. The first proposition is that, while continuity is acknowledged in parts of the literature, transition in nursing is best theorised as a continuous developmental trajectory spanning the professional lifespan, from pre‐professional identity formation through post‐retirement contribution. The second proposition is that integrating existing transition frameworks yields explanatory power beyond any single model, positioning nursing transition as a cumulative developmental process rather than a series of discrete adjustments. The third proposition is that middle‐career development constitutes an active, prolonged transition marked by transformation in practice wisdom, moral reasoning and organisational influence, yet it remains under‐theorised in transition scholarship. Underpinning these propositions is a central explanatory mechanism: transition operates as a recursive developmental process, such that adaptive capacities (e.g., identity stability, coping resources, clinical judgement) developed through one phase are carried forward and re‐used to shape adaptation in later phases. Conversely, when supports are absent, unresolved disruptions can compound across phases, for example, early identity fragility may heighten later transition shock, while sustained middle‐career reflective practice can strengthen late‐career mentorship and post‐retirement contribution.

Taken together, these propositions reposition becoming a nurse as a lifespan process in which developmental gains accumulate and shape adaptive capacity across subsequent transitions. In doing so, this framework challenges the dominant assumption in nursing scholarship that transition is a time‐limited phenomenon confined to the post‐graduation period. While the literature on new graduate transition has generated important insights into transition shock, adaptation and early attrition (Duchscher 2009; Kramer 1974), this focus has created a temporal compression that obscures how professional identity is formed long before registration and how it continues to evolve decades beyond initial employment. Reconfiguring ‘becoming a nurse’ enables greater understanding and practical solutions to real‐world challenges, such as addressing nursing workforce shortages, identifying factors that influence recruitment, retention and work satisfaction throughout evolving careers, and ways to support and foster ongoing engagement of nurses within and beyond the profession's clinical bounds.

By foregrounding early career interest formation, this analysis demonstrates that professional socialisation into nursing begins well before university enrolment, shaped by family exposure, media representations, schooling practices and early encounters with healthcare environments (Chambers et al. 2018; Price 2009; Godbey and Gordon 2019). These formative influences actively shape who sees nursing as a viable identity and career long before the formal transition to practice becomes relevant. This shifts the conceptual boundary of transition upstream and reframes recruitment not simply as a higher education issue but as a long‐term process of identity cultivation.

The conceptual contribution is further advanced by explicitly positioning second‐career entry as a structurally significant pathway into nursing rather than a deviation from traditional routes. Graduate‐entry pathways reflect broader shifts in working life, career mobility and workforce policy (Macdiarmid, McClunie‐Trust, et al. 2021). Although second‐career entrants bring considerable professional and life experience, this analysis demonstrates that their transition remains fundamentally novice in nature, characterised by identity disruption, emotional vulnerability and discipline‐specific risk (Mohamed 2019). Recognising these challenges disrupts assumptions that prior expertise inherently mitigates early nursing transition difficulties and reinforces the need for phase‐specific educational and professional supports.

This paper also reframes the middle‐career transition, often characterised in the literature as a period of consolidation or stability, as a prolonged phase of ongoing transformation, choice and evolution. The progression from competence to proficiency, expert practice and ultimately clinical wisdom is better understood as an extended developmental transition rather than a definitive endpoint (Matney et al. 2020). Professional growth in nursing unfolds continuously across the career span, with goals, motivations and developmental needs shifting in relation to role, context, interests and experience (Vázquez‐Calatayud et al. 2021). During these middle years, practice advancement reflects more than technical refinement; it encompasses the emergence of advanced moral reasoning, anticipatory judgement and increasing organisational influence (Pool et al. 2016). Recognising this sustained period of evolution as a distinct transition phase underscores the importance of targeted support to promote workforce sustainability, leadership succession, specialisation and long‐term retention (Goens and Giannotti 2024).

The inclusion of late‐career transition reframes professional exit not as termination of contribution but as a further identity transition into new forms of nursing participation. The persistence of professional identity through mentorship, governance, education and advisory roles demonstrates that becoming a nurse is not bound by employment status or participation in exclusively clinical and patient‐focused roles (MacLeod et al. 2021).

Collectively, these four phases, early career interest, non‐traditional entry, middle‐career transition and late‐career transition, constitute the Lifespan Transition Framework proposed in this paper. Rather than treating transition as episodic, the framework theorises becoming a nurse as a coherent developmental trajectory in which each phase carries distinct transition demands shaped by the interaction of identity formation, expertise development and contextual support. The following section examines how this integrated lens relates to the established transition theories on which the framework builds.

### 4.2 Relationship to Existing Transition Frameworks

The theoretical contribution of this work is not the introduction of an entirely new transition theory, but the lifespan integration of established transition and competence frameworks into a coherent explanatory model. In this synthesis, existing frameworks are treated as complementary rather than competing; each illuminates different developmental tasks (e.g., identity formation, role adaptation, capability acquisition and professional socialisation) that become more visible when traced longitudinally across the nursing career. This integrated lens clarifies why post‐graduation transition accounts are powerful yet incomplete: when frameworks are applied primarily to early career entry, they privilege short‐term adaptation and obscure longer‐range developmental dynamics. Applied across the professional lifespan, the model surfaces previously under‐articulated transition demands and informs phase‐sensitive educational and organisational responses, including upstream identity cultivation, second‐career novice identity disruption, sustained middle‐career transformation and professional identity continuity beyond employment. Rather than opposing or refuting past scholarship, we seek to build upon, synthesise and extend how it can be applied and thought about.

Schlossberg's Transition Model (Schlossberg 2005), with its emphasis on situation, self, support and strategies, has been applied extensively to undergraduate nurses but rarely to early‐career interest formation, second‐career entrants, or late‐career transition. Nevertheless, its core premise that transitions are continuous, adaptive processes shaped by personal and contextual resources remains relevant to adolescent career decision‐making, where students negotiate emerging identity, family influence, educational opportunities and sociocultural expectations. It also applies to second‐career entrants who experience significant re‐development of their professional identity, which differs from their previous career, as well as to post‐retirement professional engagement, where nurses reconfigure their identity, contribution and social connections outside formal employment.

Similarly, Kennedy's Integrated Transition Model (2018) conceptualises transition as socially constructed, dynamic and cyclical, offering particular explanatory power for understanding how individuals navigate transitions not only between undergraduate nursing student and graduate nurse roles, but also between adolescent and student, mid‐career clinician and organisational leader and employed nurse and retired mentor. This model emphasises how transitions are shaped by relational environments, professional cultures and institutional expectations. This insight is especially relevant for second‐career entrants and those from diverse cultural and linguistic backgrounds who must navigate both professional learning and sociocultural repositioning simultaneously (Dos Santos 2020; Colley et al. 2023).

Benner's Novice to Expert framework (Benner 1984), while not itself a transition theory, provides critical developmental insight into how clinical competence evolves through the middle years of practice. The progression from competent to proficient to expert, characterised by deepening tacit knowledge, refined clinical intuition and what contemporary scholarship conceptualises as clinical wisdom (Matney et al. 2020), represents a sustained form of professional transition that extends well beyond the new graduate phase. Evidence demonstrating improved system efficiency and patient outcomes among nurse practitioner hospitalists (McMenamin et al. 2023) reflects not merely advanced credentialing, but the cumulative product of long‐term reflective practice. Importantly, this expertise does not dissolve at retirement. MacLeod et al.'s (2021) finding that many nurses retain a strong professional identity post‐retirement, as evidenced in statements such as ‘First and foremost, I am a nurse’ (p. 5), indicates that expert practice transitions into new forms of contribution rather than simply ceasing.

Duchscher's Transition Shock Theory (Duchscher 2009), with its focus on the emotional, cognitive and professional dissonance experienced by new graduates, also demonstrates broader lifespan relevance when interpreted through this expanded lens. Comparable patterns of dissonance and identity disruption are evident when adolescents first confront the realities of clinical environments, when second‐career entrants relinquish established expertise to become novice nursing students, and when late‐career nurses renegotiate professional identity outside formal employment. While the situational triggers differ, the underlying adaptive processes remain consistent.

Extending these established frameworks across the professional lifespan, therefore, reveals not only how nurses adapt at specific career moments but also how transition functions as a developmental engine that shapes workforce entry, capability, retention, leadership and legacy. Crucially, this integrated application is more than synthesis. It supports a new conceptual claim: transition in nursing operates as a recursive developmental mechanism, in which adaptive capacities formed in one phase become foundational resources for navigating subsequent phases (e.g., early coping resources and identity stability supporting later adaptation; accumulated middle‐career judgement and wisdom enriching late‐career mentorship and post‐retirement contribution). When adequately supported, early novice identity disruption can build resilience that strengthens middle‐career adaptability; when structurally preserved, accumulated practice wisdom can enrich late‐career mentorship and post‐retirement contribution. This recursive quality distinguishes the Lifespan Transition Framework from a simple temporal extension of existing models and positions it as a generative conceptual tool for both theory and practice. These insights have direct implications for workforce sustainability and long‐term engagement.

### 4.3 Workforce Sustainability

When transition is understood as a lifelong professional process rather than a short‐term graduate adjustment, workforce sustainability must be approached as a whole‐of‐career strategy spanning early‐career interests, educational design, mid‐career development and late‐career retention of expertise. This comprehensive perspective is critical, as the projected global deficit of 4.1 million nurses by 2030, compounded by the imminent retirement of 19% of the current workforce (WHO 2025), cannot be addressed through isolated interventions at any single career stage.

At the upstream level, early‐career aspiration formation emerges as a critical yet underutilised workforce mechanism. With only 8% of Australian children aspiring to healthcare careers (Chambers et al. 2018), despite healthcare being the nation's fastest‐growing employment sector (Australian Government Department of Employment 2017), there is a substantial disconnection between workforce demand and early identity formation. Media portrayals and limited professional visibility continue to constrain the public image of nursing (González et al. 2023; Raymond et al. 2018). Although structured career education initiatives such as Victoria's My Career Insights program support early exploration (Victorian Department of Education 2024), nursing remains underrepresented within these platforms. Systematic engagement by practicing nurses in schools, digital media and simulation‐based outreach represents a powerful yet currently underutilised recruitment strategy.

At the midstream level, the growing reliance on graduate‐entry programs highlights second‐career recruitment as a central pillar of contemporary workforce strategy. These entrants often demonstrate high intrinsic motivation, resilience and transferable professional skills (Macdiarmid, Turner, et al. 2021; World Health Organisation 2023), yet their transitions are frequently compressed by accelerated curricula, compounded life responsibilities and profound identity disruption associated with becoming a novice in a new profession (Mohamed 2019). Without flexible learning models, meaningful recognition of prior learning and culturally responsive support structures, these pathways risk avoidable attrition (Colley et al. 2023; Dos Santos 2020; Kean et al. 2022).

The midstream workforce, importantly, is not composed solely of new entrants but also of established nurses undergoing a prolonged transition from competence to proficiency, expertise and to clinical wisdom. This phase reflects the sustained developmental trajectory articulated in Benner's work, where situated practice, pattern recognition and anticipatory judgement deepen over time (Benner 1984). Contemporary scholarship conceptualises this later‐stage development as clinical wisdom, characterised by moral sensitivity, contextual intelligence and intuitive knowing that emerges through decades of reflective engagement (Matney et al. 2020). The middle‐career transition phase is a critical period for consolidating collaborative authority, informal leadership and system influence, with experienced nurses serving as mentors, cultural carriers and safety advocates within complex healthcare environments (Gartshore et al. 2024; Sirota 2017). Without deliberate institutional investment in middle‐career development, including structured leadership pathways, advanced clinical roles, scholarly practice and protected mentorship capacity, health systems risk stagnation of expertise and erosion of future clinical and academic leadership pipelines.

At the downstream level, nurses in the late career transition phase represent a vast reservoir of clinical wisdom, leadership capacity and institutional memory. With one in six nurses expected to retire globally within the next decade, the unstructured loss of this expertise constitutes a major system vulnerability (Cimino‐Fiallos and Wolff 2022). Yet many nurses express a desire to remain professionally engaged through redesigned roles aligned with evolving physical capacity and life priorities (MacLeod et al. 2021; Denton et al. 2021a). Ergonomic redesign, flexible rostering, structured mentorship pathways, leadership recognition and post‐retirement academic and advisory roles offer tangible mechanisms for retaining expert contribution while supporting healthy workforce exit (Australian College of Nursing 2023; Denton et al. 2021b; Pagone and Briggs 2021).

Taken together, and consistent with previous work (Peters 2023), these findings demonstrate that recruitment, development, retention and post‐retirement contribution are not separate workforce challenges, but interdependent expressions of a single professional life course, requiring an integrated workforce strategy rather than isolated interventions.

## 5 Conclusion

This paper reconceptualises becoming a nurse as a continuous, lifelong transition that extends from early career interest formation through diverse entry pathways, middle‐career development and post‐retirement professional contribution. The Lifespan Transition Framework advances the field by demonstrating that transition operates as a recursive developmental mechanism across four structurally distinct yet interdependent phases, offering an integrated conceptual proposition with both explanatory and practical utility. By extending established transition frameworks across this expanded temporal frame, professional identity, expertise development and workforce sustainability emerge as cumulative and interdependent processes requiring coordinated, phase‐sensitive support throughout the career lifespan. In the context of persistent global nursing shortages and an aging workforce, this lifespan perspective provides a unifying conceptual foundation for nursing education, workforce policy and organisational practice. Rather than viewing transition as a time‐limited early‐career phenomenon, this broader understanding enables more strategic approaches to recruitment, retention and the preservation of professional wisdom across the entire nursing career.

Future research should empirically validate this lifespan framework through longitudinal studies tracking nurses from adolescence through retirement and examine how transition experiences differ across diverse populations and contexts. Intervention studies evaluating phase‐specific support strategies, from school‐based career exploration to post‐retirement mentorship models, are needed to translate this conceptual work into evidence‐based practice and inform sustainable workforce planning.

## Funding

The authors have nothing to report.

## Ethics Statement

This manuscript is an analytical discursive paper and does not report primary research involving human participants, identifiable personal data, or patient records. Therefore, formal ethics approval was not required.

## Conflicts of Interest

Authors Seung A (Sarah) Park, Marion Eckert, Rebecca Sharp, Judy Duchscher and Micah DJ Peters declare no commercial affiliation in relation to the work underpinning this manuscript nor in relation to the preparation of the manuscript. The attached manuscript is the original work of the authors and has not been previously published or under consideration for publication elsewhere.

## Data Availability

The authors have nothing to report.
